# Evaluation of Saliva as a Matrix for RT-PCR Analysis and Two Rapid Antigen Tests for the Detection of SARS-CoV-2

**DOI:** 10.3390/v14091931

**Published:** 2022-08-30

**Authors:** Julie De Meyer, Hanne Goris, Olivier Mortelé, An Spiessens, Guy Hans, Hilde Jansens, Herman Goossens, Veerle Matheeussen, Sarah Vandamme

**Affiliations:** 1 Department Microbiology, Laboratory Medicine, Antwerp University Hospital (UZA), 2650 Edegem, Belgium; 2 Mobile Testing Team, Antwerp University Hospital (UZA), 2650 Edegem, Belgium

**Keywords:** SARS-CoV-2, saliva, rapid antigen test

## Abstract

The use of saliva for the detection of severe acute respiratory syndrome coronavirus 2 (SARS-CoV-2) sparks debate due to presumed lower sensitivity and lack of standardization. Our aim was to evaluate the performance characteristics of (i) saliva collected by the ORAcollect^TM^ device as a matrix for SARS-CoV-2 reverse-transcriptase polymerase chain reaction (RT-PCR), and (ii) 2 saliva rapid antigen tests (AgRDT). From 342 ambulatory individuals, both a nasopharyngeal swab and saliva sample via ORAcollect^TM^ were obtained for a SARS-CoV-2 RT-PCR test. Furthermore, 54 and 123 additionally performed the V-Chek^TM^ or Whistling^TM^ saliva AgRDT. In total, 35% of individuals screened positive for SARS-CoV-2 via nasopharyngeal swab. Saliva, as a matrix for the RT-PCR, had a specificity of 96.5% and a negative predictive value (NPV) of 91.3%. Interestingly, 6 out of 8 patients thought to be false positive in saliva re-tested positive by nasopharyngeal sampling after 2 to 9 days. Both V-Chek^TM^ and Whistling^TM^ AgRDT had a lack of sensitivity, resulting in an NPV of 66.9 and 67.3%, respectively. Saliva proved to be a sensitive and specific matrix for SARS-CoV-2 detection by the RT-PCR. In this setting, saliva might have an earlier window of detection than the nasopharyngeal swab. By contrast, both AgRDT showed an unacceptably low sensitivity and NPV.

## 1. Introduction

In 2019, a new coronavirus emerged in Wuhan, China which was later named severe acute respiratory syndrome coronavirus 2 (SARS-CoV-2) [1]. The outbreak was declared the 6th public health emergency of international concern by the World Health Organization (WHO) on the 30th of January 2020 [2] and is still a global health concern. A need for reliable and rapid SARS-CoV-2 detection methods arose in order to limit the spread of the virus. A reverse-transcriptase polymerase chain reaction (RT-PCR) on a nasopharyngeal specimen became the gold standard for COVID-19 diagnostics because of its high sensitivity and specificity. However, trained personnel and specialized, expensive equipment is required for this method and the sample taking is often experienced as unpleasant by the patient. Therefore, accessible, affordable, and reliable self-assessment methods were needed, which led to the development of antigen rapid diagnostic tests (AgRDT). These devices are mainly lateral flow immunochromatography-based point-of-care tests [3] with a short turn-around-time (TAT), although with lower sensitivity and specificity in comparison to the golden standard RT-PCR on a nasopharyngeal swab [4,5].

Saliva as a specimen for COVID-19 testing has been suggested, specifically in combination with an RT-PCR [6,7]. Yet, the literature has been ambiguous about the sensitivity of saliva AgRDT and hence the reliability [5,7]. A recent study reported an increased sensitivity in patients with a medium to high viral load, i.e., the most contagious patients [8]. The latest SARS-CoV-2 variant of concern (VOC), omicron, is suspected of higher viral shedding in saliva in comparison to nasal samples [9], which could be due to an altered tissue tropism, i.e., a tropism for the bronchus instead of the lung [10]. This could imply that a different test strategy is required for the newest VOC. In this monocentric study, two rapid antigen tests based on saliva and an RT-PCR based on a saliva swab were evaluated in comparison to the RT-PCR on a concomitant nasopharyngeal swab. At the time of the study (16 December 2021–7 January 2022), the omicron BA.1 variant was emerging in Belgium.

## 2. Materials and Methods

### 2.1. Patients

At the testing site of the University Hospital of Antwerp, outpatients presented themselves for RT-PCR testing via nasopharyngeal swab according to the indications of the national testing strategy issued by the Belgian government. Between the 16th of December 2021 and the 7th of January 2022, patients were recruited voluntarily to participate in this study. In total, 342 patients donated saliva via the ORAcollect^TM^ swab (DNA Genotek Inc., Ottawa, ON, Canada). Among them were 36 children (10.4%), with the consent of their parents. Five patients were tested twice in this period resulting in a sample size of 347 saliva samples. The age range was 3–72 years (median age 30 years), of which 193 (55.6%) were female. On top of the ORAcollect^TM^ swab, 123 patients performed the Whistling^TM^ 2019-nCoV Saliva Ag Easy Test (Guangzhou Decheng Biotechnology CO., LTD, Guangzhou, China), among which there were 6 children (11.1%) and 33 women (61.1%). The age range was 7 to 63 years with a median age of 31 years. Additionally, 54 unique patients performed the V-Chek^TM^ 2019-nCoV Saliva Rapid Test Card (Lollipop Test) (Guangzhou Decheng Biotechnology CO., LTD). Furthermore, 11 of these patients were children (8.9%) and 69 were female (56.1%). The age ranged from 9 to 72 years (median age 39 years). Ethical approval was by the Ethical Committee of the University Hospital of Antwerp (B3002020000171).

### 2.2. SARS-CoV-2 Detection Methods

#### 2.2.1. Real-Time PCR Using Nasopharyngeal Swabs

Respecting preventive safety measures, samples were collected by qualified personnel in sample collection tubes with an InActiv Blue^®^ virus-inactivating and RNA-stabilizing transport medium (Fertipro NV, Beernem, Belgium). The virus was further inactivated by incubation for 30 min at 74 °C. After vortexing the sample, 200 µL of transport medium was transferred into a 96 Deepwell plate (Thermo Fisher Scientific^TM^, Waltham, Massachusetts, USA) and the bacteriophage MS2, a single stranded RNA virus, was added as the internal control. RNA was extracted using the MagMAX™ Viral/Pathogen II Nucleic Acid Isolation Kit on the KingFisher^TM^ Flex Purification System. The TaqPath™ COVID-19 CE-IVD RT-PCR kit (Thermo Fisher Scientific^TM^, Waltham, Massachusetts, USA) was used for the PCR in the QuantStudio^TM^ 5 instrument (ThermoFisher). This assay is designed to detect three SARS-CoV-2 target genes: ORF1ab, S, and N. FastFinder software (v4.6.3, UgenTec, Hasselt, Belgium) was used for the analysis and interpretation of the results.

#### 2.2.2. Real-Time PCR Using ORAcollect^TM^

**Figure 1:**
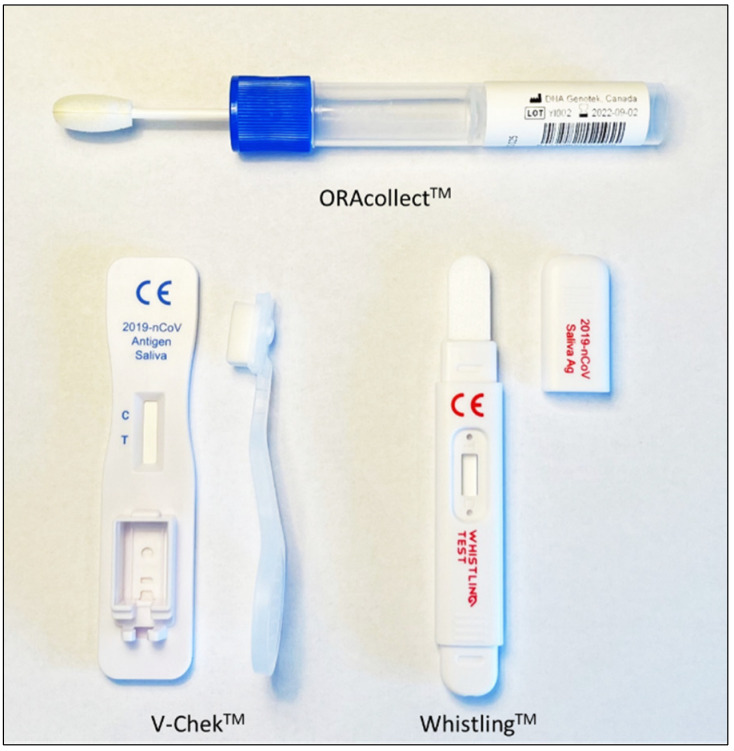
Overview of the three tests: the ORAcollect^TM^ saliva swab as a screw cap on the pre-barcoded tube containing virus inactivating medium (**top**), the V-Chek^TM^ test card and saliva swab (**bottom left**), and the Whistling^TM^ midstream test with absorbent tip for saliva (**bottom right**).

After ensuring that patients had not eaten, drunk, or smoked 30 min prior to the sampling, patients were instructed on how to collect their own saliva samples according to the instructions from the kit insert (DNA Genotek Inc., Ottawa, ON, Canada). Sample collection was supervised by trained personnel. The ORAcollect^TM^ swab (Figure 1) was put between the left cheek and teeth and was rotated 10 times. This procedure was repeated on the right side with the same swab, before putting it into the virus-inactivating medium and inverting the sample 15 times. The maximum uptake of the swab was estimated at 500 to 550 µL. Then, 350 µL of sample was used for extraction using the same kit as described above, while the extraction program was adjusted to the saliva and its differing volume, using a protocol with longer binding and drying steps, and a higher binding temperature but lower elution temperature. The SARS-CoV-2 RT-PCR was performed with the same method and kit as described above.

#### 2.2.3. AgRDT Using V-Chek^TM^ and Whistling^TM^ Test

Both lateral flow immunoassays V-Chek^TM^ 2019-nCoV Saliva Rapid Test Card and Whistling^TM^ 2019-nCoV Saliva Ag EASY TEST (Figure 1) qualitatively detect the nucleocapsid protein antigen of SARS-CoV-2. Both tests are marketed for professional and layperson’s use. Patients were not allowed to drink, eat, or smoke 30 min prior to the test procedure. Sample collection was supervised by trained personnel. Patients were instructed to swab the inside of the mouth including the tongue for 90 s using the V-Chek^TM^, ensuring the indicator turned blue which meant enough saliva was absorbed into the sponge, and to put the test swab into the test card holder. The maximum uptake of the sponge was estimated at 1000 µL. In the case of the Whistling^TM^ test, patients were instructed to put the test strip under the tongue until the purple color moved across the result window. The maximum uptake of the test strip was estimated at 750 µL. The result of the tests was interpreted after 10 min according to the manufacturer’s instructions based on the appearance of lines on the test and control positions.

### 2.3. Statistical Analyses

The results of the RT-PCR on the nasopharyngeal swab were used as the gold standard [11] for comparison. The sensitivity, specificity, negative predictive value (NPV), and positive predictive value (PPV) were calculated for all three methods. The test agreement between the nasopharyngeal RT-PCR, ORAcollect^TM^ RT-PCR, V-Chek^TM^, and Whistling^TM^ test was assessed using Cohen’s κ concordance testing [12]. A chi-square (chi^2^) test was performed to determine whether there were differences between subgroups in the test population, with a P value < 0.05 considered as statistically significant. Differences in cycle threshold (Ct) values between methods were visualized using Bland–Altman plots and a whisker box plot. Microsoft Office Excel^®^ 2016 software (Microsoft Corporation, Redmond, WA, USA) was used for all statistical analyses described above.

## 3. Results

### 3.1. ORAcollect^TM^ Compared to Nasopharyngeal Sample

**Figure 2:**
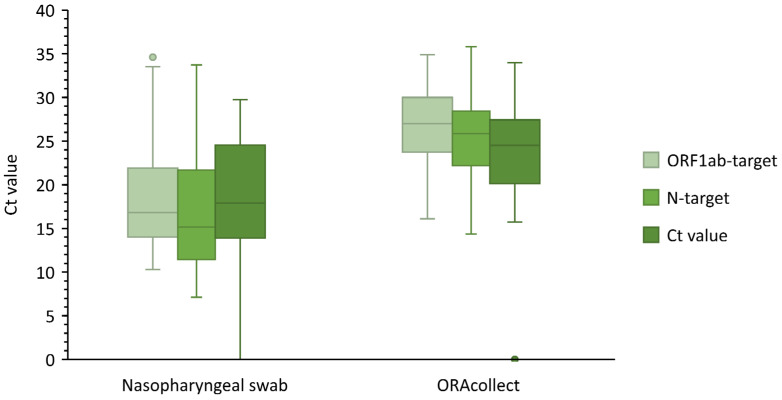
Whisker box plot comparing Ct values obtained by RT-PCR from nasopharyngeal swab and ORAcollect^TM^ saliva swab.

The positivity ratio of nasopharyngeal swabs in our study population was 34.9%, of which 87.6% showed S gene target failure (presumably omicron). A small subset of samples (3.5%) was sequenced by whole genome sequencing (WGS) in the context of baseline surveillance. Each of the 12 sequenced samples turned out to be the BA.1 subvariant (B.1.1.529 lineage). An analysis of the 347 nasopharyngeal and saliva samples led to 318 concordant results (91.6%). In total, 100 paired samples tested positive for both matrices, while 218 paired samples tested SARS-CoV-2 negative (Table 1). Furthermore, 29 (8.4%) discordant results were observed between the use of a nasopharyngeal swab and saliva. Eight patients tested positive by the ORAcollect^TM^ method, but negative with the nasopharyngeal swab, and were presumed to be false positive. However, in six cases, the patients did test positive by nasopharyngeal swab when tested again (2–9 days later). The remaining two patients were not retested. The RT-PCR using saliva as a matrix was not able to detect SARS-CoV-2 in 21 samples that were SARS-CoV-2-positive by nasopharyngeal swab (Ct _N-target_ 10.4–33.5, µ = 25.4). Twelve of those nasopharyngeal samples had Ct_N-target_ values above 27.6 (cutoff by the executive laboratory to define weakly positive samples). There was no difference in discordance between children and adults (P = 0.98) or between different test indications (P = 0.79) according to the performed chi^2^ test. The ORAcollect^TM^ method showed a sensitivity of 82.6% and specificity of 96.5%. The Cohen’s concordance was very good (κ = 0.81), and the PPV and NPV were 92.6% and 91.3%, respectively. A whisker box plot (Figure 2) was constructed to visualize the average Ct values of both methods. The result shows that the ORAcollect^TM^ method has a significantly higher average Ct value (P = 0.003) in comparison to the concomitant nasopharyngeal swab, despite using a higher input volume for extraction. It is important to note that the whisker of the S-target is as low as zero due to the samples displaying an S-gene target failure.

Bland–Altman plots were constructed for all three tested targets (Figures S1–S3), presenting the difference in Ct value (ΔCt) versus the average of analyses for both sample types. For example, for the N-target, the average ΔCt between both methods was 9.9 (95% CI [−2.3, 20.5]). Discordant results were excluded for this analysis.

### 3.2. V-Chek^TM^

Out of 54 patients, 1 was positive for both tests and 37 were negative for both tests (Table 1). The test was invalid in four cases (7.4%). False negative results were noted for 12 patients (22.2%), with a significantly higher discordancy in children (age < 18, P = 0.035). The only individual who tested strongly positive on the nasopharyngeal PCR (Ct _N-target_ ≤ 9.98) and on the saliva sample (Ct _N-target_ ≤ 14.81) was detected by the V-Chek^TM^ test. Hence, the V-Chek^TM^ showed a sensitivity of 7.7%, an NPV of 66.9%, and a specificity and PPV of 100%. The concordance between both methods was low (κ = 0.12).

### 3.3. Whistling^TM^ Test

Out of 123 patients, 5 were positive and 47 were negative by both methods (Table 1). The test was invalid for 21 patients (17.1%), and 50 patients showed false negative results (40.7%) leading to a very low concordance (κ = 0.07). There was no observed difference in discordancy or invalidity levels between adults and children (P = 0.09 and P = 0.91, resp.). Only individuals who tested strongly positive on the nasopharyngeal PCR were detected by the Whistling^TM^ test (Ct _N-target_ ≤ 21.4). These individuals tested strongly positive on the saliva sample as well (Ct _N-target_ ≤ 23.4). A sensitivity of 9.1% and a specificity of 100% were found for the Whistling^TM^ test. The PPV and NPV were, respectively, 100% and 67.3%.

## 4. Discussion

A rapid diagnosis of COVID-19 infection can be important to help limit the viral spread. Using saliva as an alternative for a nasopharyngeal swab for the detection of COVID-19 has been suggested in the literature. Mestdagh et al. found that samples obtained by spitting have a higher sensitivity in comparison to swabbing, but both are lower than nasopharyngeal samples [8]. Two rapid antigen tests and one PCR test based on saliva were tested in comparison to the PCR on nasopharyngeal samples.

Saliva-based diagnostic methods provide some advantages over nasopharyngeal samples; sample collection is painless and can be performed by non-healthcare professionals, making self-sampling possible. This reduces the risks of virus transmission to healthcare workers conducting the sampling [13] as the collection of nasopharyngeal swabs may induce sneezing, coughing, and expelling virus particles. The reduced risk of infection to healthcare staff can lower the need for personal protective equipment.

Rapid antigen tests (e.g., V-Chek^TM^ and Whistling^TM^ test) have a short TAT (10 min), are easy to use, and do not require any laboratory environment. Therefore, they would be very useful as point-of-care tests and would be preferred in large screenings, e.g., in schools. School children could perform such tests themselves on a regular basis in order to obtain a baseline screening. In addition, in circumstances where the same people are living closely together over longer periods of time (e.g., homeless shelter, long-term care facilities) this can be of added value. However, the reported sensitivity and NPV for both self-assessment methods in this study were very low in comparison to the gold standard and to what the manufacturer promised (V-Chek^TM^: sensitivity 95.7%, specificity 98.4%; Whistling^TM^ test: sensitivity 93.9%, specificity 99.2%, according to the manufacturers). Both kit inserts reported a limit of detection of 100 tissue culture infectious doses (TCID_50_) per mL as corroborated by our findings. Saliva rapid antigen testing was soon halted in this study given the disappointing results. Furthermore, both tests showed a high invalidity rate, which might be due to interpatient differences such as a higher viscosity of the saliva which impeded the strip from absorbing the saliva properly. The higher invalidity in children could be explained by the incorrect or insufficient execution of the test by the patient, though this can also be the case for adults. However, as children represented only 10% of the study population, a statistically significant difference could not be reached. Yet, according to the presented data, we would not recommend these saliva rapid antigen tests as they lacked sufficient accuracy for routine use. The lower diagnostic performance might be explained by the lower viral load in saliva as compared to nasopharyngeal swabs as found by Kritikos et al. [14], or the presence of mucosal secretory immunoglobulins targeting SARS-CoV-2 antigens and thus competing with an AgRDT for the same target [15,16]

The European Centre for Disease Prevention and Control previously suggested that rapid antigen detection tests should be used as complementary to molecular diagnostics and are not sufficient when used alone [7]. Khalid et al. found that among rapid antigen detection tests, saliva tests have the lowest sensitivity [5]. However, the study by Khalid et al. was performed before the emergence of the omicron variant, whereas our study was performed during a period when the omicron BA.1 emerged.

ORAcollect^TM^ has the advantage of being a less invasive method in comparison to the nasopharyngeal swab, but its TAT is similar to the gold standard and still requires a laboratory setting, trained personnel, and specialized instruments. The method has an acceptable sensitivity (82.6%) and specificity (96.5%) within the promised range from the manufacturer [17]. A rather large gap in Ct values was observed between both methods. When a larger volume (350 µL vs. 200 µL) was used for the extraction of ORAcollect^TM^ samples, ΔCt became smaller (data not shown), but this often did not leave enough samples for potential re-analysis [18]. Another option to minimize the ΔCt could be the further optimization of the pre-analytic phase, for example, a color change of the saliva swab to indicate a sufficient amount of saliva was absorbed.

For 29 patients, the results were discordant. Of the eight patients with a presumed false positive result using the ORAcollect^TM^ method, six patients did test positive by a nasopharyngeal swab 2 to 9 days later. S-gene target failure was detected for all six patients, possibly indicating the presence of the omicron BA.1 variant. Marais et al. previously reported an increased viral shedding in saliva in comparison to the nasopharynx, regarding omicron infections [9]. This could be an explanation for the discordant results and would suggest that saliva could possess an earlier window of detection. However, not enough data were gathered to test the difference in detection between different VOCs. The other false positive cases were not retested; thus, further assessment was not possible. A false negative result was observed in 21 patients, of which 12 were weakly positive on nasopharyngeal swabs. False negative samples that were actually clearly positive by a nasopharyngeal swab (*n* = 9) highlighted the importance of the pre-analytic phase, as this might be due to an insufficient amount of saliva taken up by the device. An assay with RNAseP, an internal human control target, was performed to test whether faulty sampling could be the cause of the discordancy. However, RNAseP was detected in all but one (nasopharyngeal) sample (data not shown), and thus cannot explain the differences between the nasopharyngeal samples and the ORAcollect^TM^ samples [19]. Another explanation could be that the time interval between sampling and last food or drink intake was not respected, despite the instructions, as was the case for two false negative patients in the study of Jegerlehner et al. [20].

In this study, ORAcollect^TM^ samples showed significantly higher Ct values (P = 0.003) for all three tested target genes, compared to those from the nasopharyngeal swabs. This is in accordance with the findings of Pasomsub et al. who reported higher median Ct values of the ORF1ab and N genes in saliva specimens compared to nasopharyngeal and throat specimens [21]. Saliva is a complex material shown to contain SARS-CoV-2 specific antibodies [22], and different viral kinetics compared to nasopharynx, which might explain the difference in Ct values [23]. ORAcollect^TM^ did not detect some weakly positive samples, which can be explained by the findings of Mestdagh et al. that the ORAcollect^TM^ method has a higher sensitivity in patients with a medium to high viral load [8]. The latter sparks the discussion of whether it is clinically relevant to detect these weakly positive patients as they may not be contagious due to a lower viral load [24,25].

It is suggested that early morning saliva specimens, before tooth brushing, using mouthwash, and eating, are more likely to have a higher viral load and thus present with a greater sensitivity in comparison to saliva samples collected later in the day [26,27,28]. Therefore, the timing of the sample collection could influence the test result. Our study was limited in this way, as patients were tested at any time of day and at differing time points after high-risk contact or in the course of their infection. This might affect the diagnostic accuracy of saliva and nasopharyngeal swab testing differently. Yet, as saliva testing might be interesting for large screening purposes, a real-life situation was mimicked, adding robustness to our findings.

Furthermore, the presented study was limited regarding the study population, being defined by the implemented test strategy at the time and by the testing location. For example, hospitalized patients and children were underrepresented, and thus our findings may not be generalized. To the extent possible, our results were checked for potential differences in age and different test indications.

The main strength of this study was evaluating the performance of saliva testing not only via PCR, but also via rapid antigen testing, and the comparison with a concomitant nasopharyngeal swab. Additionally, it was checked if patients were re-tested within a few days to intercept possible false negatives or false positives. Lastly, this study addressed a clinically relevant topic that could affect public health policies for testing strategies.

Once the above-described results were obtained, the use of ORAcollect^TM^ sampling was introduced in highly regulated settings such as institutions where needy young people, e.g., the mentally disabled, stayed during the week. After a first infection was detected in such residential groups, saliva sampling was performed three times a week (in the morning) to quickly detect further infections within the group.

## 5. Conclusions

Saliva proved to be a sensitive and specific sample type for SARS-CoV-2 detection by RT-PCR. Furthermore, the data suggest that saliva has an earlier window of detection for the omicron BA.1 subvariant, which could be a major advantage. However, more data are needed to compare the detection of different variants of concern. Both saliva rapid antigen tests showed an unacceptably low sensitivity and are therefore not recommended to be implemented as a screening tool during the COVID-19 pandemic.

## Figures and Tables

**Table 1:** Overview of the results for each saliva method compared to the nasopharyngeal swab.

	ORAcollect^TM^ (RT-PCR)	V-Chek^TM^ (AgRDT)	Whistling Test^TM^ (AgRDT)
True positives	100 (28.8%)	1 (1.9%)	5 (4.1%)
True negatives	218 (62.8%)	37 (68.5%)	47 (38.2%)
False positives	8 (2.3%)	0 (0.0%)	0 (0.0%)
False negatives	21 (6.1%)	12 (22.2%)	50 (40.7%)
Invalid results	0 (0.0%)	4 (7.4%)	21 (17.1%)
Total number of samples	347	54	123
Sensitivity	82.6%	7.7%	9.1%
Specificity ^b^	96.5%/99.1% ^b^	100%	100%
NPV ^a^	91.3%	66.9%	67.3%
PPV ^a,b^	92.6%/98.0% ^b^	100%	100%

^a^ NPV and PPV were calculated based on a disease prevalence of 34.8%; ^b^ the recalculated values for specificity and positive predictive value (PPV) after correction of the data for the 6 false positive cases who retested positive for SARS-COV-2 by nasopharyngeal sampling within 2–9 days.

## Data Availability

The data presented in this study are available on request from the corresponding author. The data are not publicly available due to privacy restrictions.

## Supplementary Materials

The following supporting information can be downloaded at: https://www.mdpi.com/article/10.3390/v14091931/s1, Figure S1: Bland–Altman plot for the N-target; Figure S2: Bland–Altman plot for the ORF1ab-target; Figure S3: Bland–Altman plot for the S-target.
