# Supplementary material for: Evaluation of Saliva as a Matrix for RT-PCR Analysis and Two Rapid Antigen Tests for the Detection of SARS-CoV-2
Source: Viruses. 2022 Aug 30;14(9):1931. doi: 10.3390/v14091931 (PMC9502549; doi:10.3390/v14091931)
Supplement: Supplementary file 1 [file viruses-14-01931-s001.zip › viruses-1888906-supplementary.pdf]

Supplementary materials

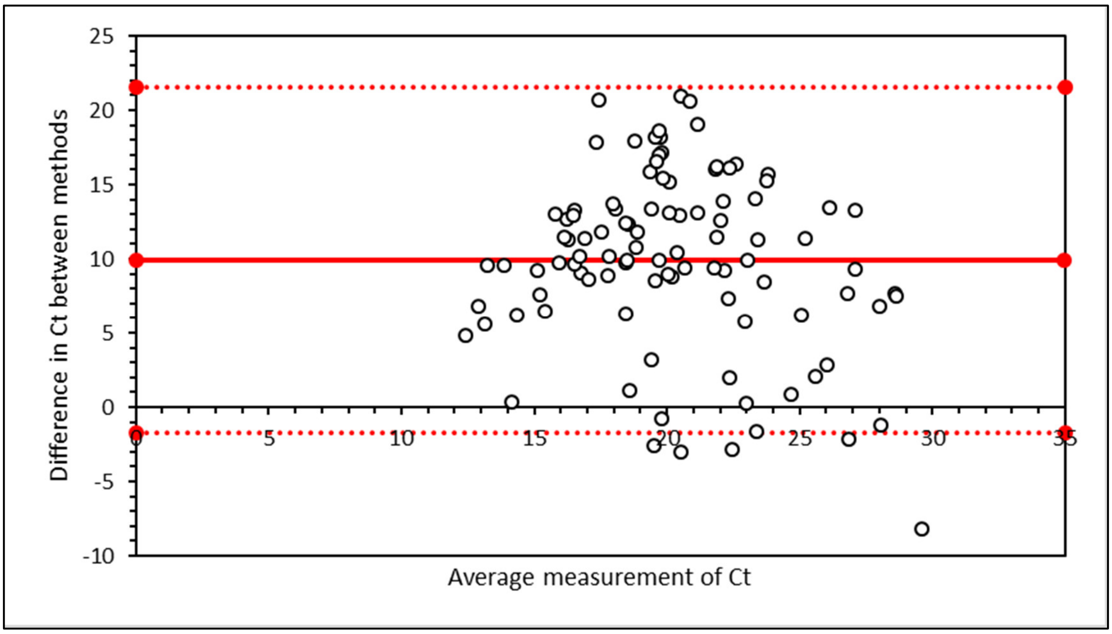

Figure S1 Bland-Altman plot of Ct values measured for the *N* target in ORAcollect samples versus nasopharyngeal samples for samples with concordant results. The full line represents the average difference measured between both methods (9.9 Ct) and the dotted lines represent the 95% confidence limits [-2.3, 20.5].

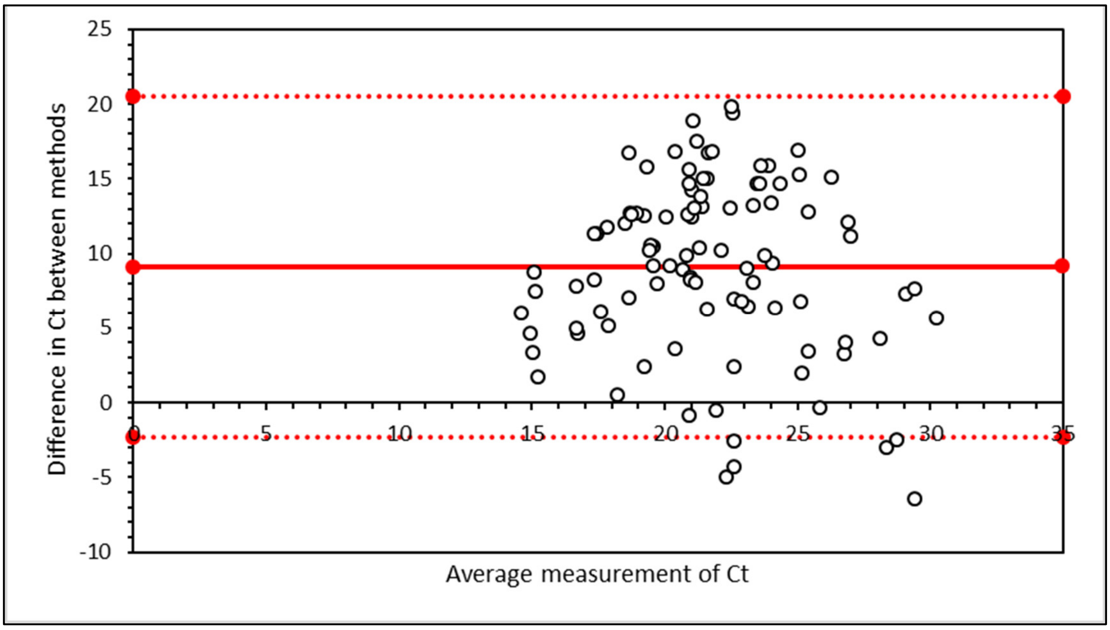

Figure S2 Bland-Altman plot of Ct values measured for the *ORF1ab* target in ORAcollect samples versus in nasopharyngeal samples with concordant results. The full line represents the average difference measured between both methods (9.1 Ct) and the dotted lines represent the 95% confidence limits [-1.7, 21.6].

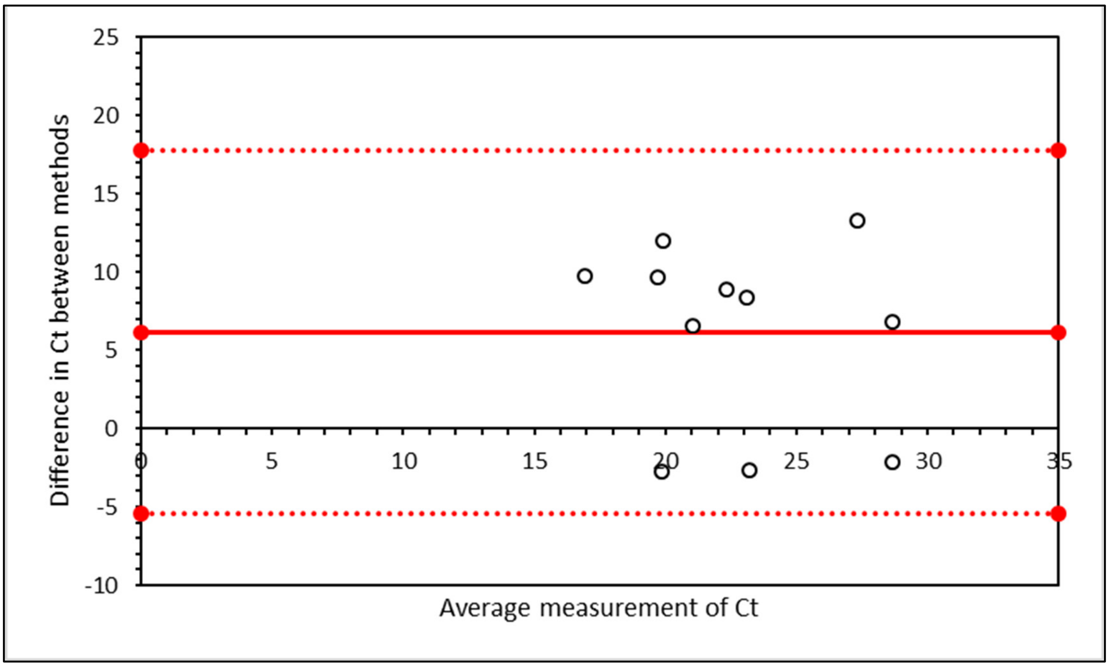

Figure S3 Bland-Altman plot of Ct values measured for the *S* target in ORAcollect samples versus in nasopharyngeal samples with concordant results. The full line represents the average difference measured between both methods (6.2 Ct) and the dotted lines represent the 95% confidence limits [-5.4, 17.7].
